# External Validation of an Open-Source Model for Automated Muscle Segmentation in CT Imaging of Cancer Patients

**DOI:** 10.3390/jimaging12030135

**Published:** 2026-03-18

**Authors:** Hendrik Erenstein, Jona Van den Broeck, Annemieke van der Heij-Meijer, Wim P. Krijnen, Aldo Scafoglieri, Harriët Jager-Wittenaar, Martine Sealy, Peter van Ooijen

**Affiliations:** 1 Department of Medical Imaging and Radiation Therapy, Hanze University of Applied Sciences, 9714 CA Groningen, The Netherlands; a.van.der.heij-meijer@pl.hanze.nl; 2 Department of Radiotherapy, University of Groningen, University Medical Centre Groningen, 9713 GZ Groningen, The Netherlands; p.m.a.van.ooijen@umcg.nl; 3 Research Group Healthy Ageing, Allied Health Care and Nursing, Hanze University of Applied Sciences, 9747 AC Groningen, The Netherlands; w.p.krijnen@pl.hanze.nl (W.P.K.); ha.jager@pl.hanze.nl (H.J.-W.); m.j.sealy@pl.hanze.nl (M.S.); 4 Experimental Anatomy Research Group, Department of Physiotherapy, Human Physiology and Anatomy, Faculty of Physical Education and Physiotherapy, Vrije Universiteit Brussel, 1050 Brussels, Belgium; jona.van.den.broeck@vub.be (J.V.d.B.); aldoscafoglieri@yahoo.com (A.S.); 5 Bernoulli Institute for Mathematics, Computer Science and Artificial Intelligence, University of Groningen, 9700 AK Groningen, The Netherlands; 6 Radboud university medical center, Department of Gastroenterology and Hepatology, Dietetics, 6525 GA Nijmegen, The Netherlands; 7 Data Science Center in Health (DASH), University Medical Centre Groningen, 9713 GZ Groningen, The Netherlands

**Keywords:** muscle segmentation, artificial intelligence, computed tomography, nnU-Net, SAROS

## Abstract

Computed tomography (CT) at the third lumbar vertebra (L3) is widely used for muscle quantification, but manual segmentation is labor intensive. This study externally validates an AI model, trained on a public dataset, for automated L3 muscle segmentation using an independent cohort, including a subgroup analysis of subject characteristics (e.g., age and a history of cancer). The AI model was trained on 900 CT scans with expert annotations from a publicly available repository. Validation was performed on 232 PET CT scans from the University Hospital Brussels, each manually segmented by an expert. Segmentation post-processing employed a density-based clustering algorithm to discard arm muscles and Hounsfield unit (HU) thresholding to refine the muscle segmentation. Performance was assessed using the Dice Similarity Coefficient (DSC) and Segmentation Surface Error (SSE). The model achieved a median DSC of 0.978 and a median SSE of 3.863 cm^2^ across the validation set. At lower BMI values, the model was more prone to overestimation of muscle surface area. Most segmentation errors occurred in the abdominal wall muscles. Analysis showed no significant difference between arm positioning above the head and alongside the body, indicating robustness to minor artifacts from arm positioning. The AI model delivers accurate, automated L3 muscle segmentation, supporting larger-scale body composition studies. However, diminished accuracy at low BMI values and limited demographic diversity of the data highlight the need for broader validation.

## 1. Introduction

Sarcopenia is characterized by loss of muscle mass and is a multi-faceted problem in the aging population. Individuals affected by sarcopenia often experience reduced quality of life as it limits self-reliance and increases both recovery times and the risk of falls [1,2].

The well-being of individuals with low muscle mass can be enhanced through the implementation of personalized (p-)rehabilitation programs. These programs are designed to be implemented either before or after medical interventions and combine tailored nutritional therapy with targeted physiotherapy (p-)rehabilitation strategies to increase muscle mass [3,4]. To enable such personalized approaches, muscle mass is often quantified using surface area measurements to identify individuals with low muscle mass [2,5,6,7]. A reference standard for muscle quantification is the segmentation of trunk muscles using computed tomography (CT) images at the third lumbar vertebra (L3) [1,5,6,8,9,10,11]. Muscle segmentation from CT scan images is often performed opportunistically, leveraging scans acquired for other clinical purposes [12]. However, manual muscle segmentation is a labor-intensive process and requires training. Consequently, despite the availability of CT images, manual CT-based muscle segmentation is often constrained. Artificial Intelligence (AI)-driven muscle segmentation can substantially reduce the labor-intensive manual tracing that currently limits the routine use of CT-based muscle quantification [13,14,15,16,17,18,19,20]. Two recent studies that employed commercial AI platforms reported high agreement with manual segmentation, with Jaccard indices of 97.74% (Dietz et al.) and Dice Similarity Coefficients (DSCs) of 0.99 (Dabiri et al.) [14,19]. While these figures demonstrate near-perfect overlap, the reliance on proprietary software introduces cost barriers that may restrict access. In contrast, several open-source or academic efforts for muscle segmentation report a more modest performance, typically DSC < 0.95 [15,16,17,18,20]. As highlighted in the systematic review by El-Kahim et al. [13], external validation is essential before a method can be trusted for a broad application. Unfortunately, many published AI-based segmentation studies either omit detailed subject characteristics (e.g., sex and age) or neglect external validation. Moreover, the development of custom segmentation tools demands large, expertly annotated training sets and specialized AI expertise. Therefore, the development of these tools remains resource-intensive which further limits widespread adoption.

Pre-built AI workflows can be used to address challenges related to AI development, such as those concerning data and expertise. An example of such a workflow is named nnU-Net, which automatically adapts to new segmentation tasks [21,22]. Additionally, the release of a dataset, named SAROS, containing 20,150 annotated CT slices is a promising source for the training of an AI model aimed at muscle segmentation [23,24]. The integration of nnU-Net and SAROS has the potential to facilitate low-cost, AI-based muscle segmentation, thereby enhancing accessibility for clinicians and researchers.

The aim of this study was to externally validate an AI model, trained using nnU-Net and the SAROS dataset, for the automated segmentation of muscles at the L3 vertebral level on CT images acquired for cancer imaging purposes.

## 2. Materials and Methods

The AI model adopted in this project was trained using the nnU-Net workflow and the SAROS dataset. Validation was conducted on independently collected clinical data acquired during the cancer diagnostic pathway, serving as an external benchmark. The segmentations generated by the model were processed using a density-based clustering algorithm (DBSCAN) and HU thresholding. Accuracy was primarily assessed by the DSC and muscle Segmentation Surface Error (SSE in cm^2^), which were further analyzed to evaluate performance across different subject characteristics.

### 2.1. Model and Training Data

**Figure 1:**
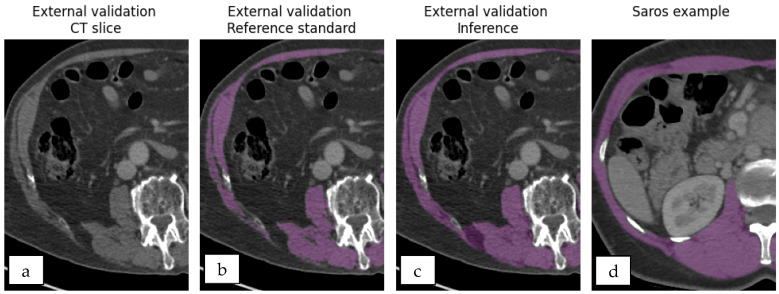
External validation (**a**–**c**) and SAROS (**d**) examples highlight the need for HU thresholding in segmentation (purple). Model segmentations without thresholding (**c**) lack the detail present in the reference standard (**b**), reflecting the limited detail inherent in SAROS data (**d**).

The data used in training nnU-Net during this study are the heterogeneous SAROS (version 2) open dataset, sourced from The Cancer Imaging Archive (TCIA) [24]. The dataset combines several pre-existing databases from TCIA, resulting in a heterogeneous collection characterized by diverse oncological profiles, varying use of intravenous (IV) contrast, and differences in CT and PET-CT acquisition protocols. SAROS includes abdominal (*n* = 300; 150 male, 150 female), thoracic (*n* = 300; 145 male, 138 female), and whole-body scans (*n* = 299; 149 male, 150 female). Semantic segmentations on numerous organs and body regions are present at every fifth axial slice, resulting in a total of 20,157 annotated slices. An example of muscle segmentation is compared to other segmentations in Section 2.3, Figure 1. All slices were included during the training process.

Sparse segmentation, every fifth slice, was implemented to reduce segmentation time and permit experienced human readers to make manual corrections. More details on the dataset are available in the original publication by Koitka et al. and the corresponding TCIA repository [23,24]. All data were processed in accordance with the TCIA restricted license agreement, complying with the ethical guidelines. All SAROS CT slices and corresponding muscle segmentations were presented to the nnU-Net, as described by the developers of nnU-Net [22]. To ensure reproducibility, the original data were not manipulated, and model training was performed according to the nnU-Net implementation guidelines [21,22].

Based on the guidelines described by the original authors, Isensee et al., a 2D model was trained, adopting five-fold cross-validation predefined by SAROS [21]. Each fold adopted an Adam optimizer with an initial learning rate of 3 × 10^−4^, consisting of 720 training and 180 validation cases, and ran for 1000 epochs. For more technical details we refer to the original publication by Isensee et al.

### 2.2. Reference Standard for External Validation

External validation data used as the reference standard were retrospectively obtained from the Radiology Department of the University Hospital Brussels, with the collection and verification performed by an experienced radiologist [5,6,7]. Data collection was approved by the Brussels Medical Ethics Commission with B.U.N. 143201942-468. The study population encompassed subjects over 18 years old who received a diagnosis of one of the following four types of tumors between 2014 and February 2021: head and neck, esophageal, lung, or melanoma. Subjects with confirmed cancer diagnosis within two years prior to the diagnostic scan, or with known metastases, were excluded from the study. PET-CT scans were acquired during each subject’s diagnostic pathway on one of three different devices: Philips GEMINI TF TOF 64, Siemens Biograph 20, and Siemens Biograph 128. Regardless of the device used, all CT scans were acquired using a peak voltage of 120 kilovolt (kV) and a slice thickness of 2 mm.

Additional scanning information was collected to evaluate potential confounding factors. The use of IV contrast was included as a factor due to its possible impact on muscle image quality. In addition, the factor of arm positioning alongside the body was included, as it can introduce streak artifacts that may affect model performance.

The following (co-)variables from the electronic patient records were included: sex, age, body mass index (BMI), and Charlson Comorbidity Index (CCI). BMI (kg/m^2^) was calculated as body weight (kg)/height^2^ (m^2^). Individuals were classified as underweight using criteria established by the Global Leadership Initiative on Malnutrition (GLIM). Subjects were considered underweight with a BMI below 20 kg/m^2^ and aged <70 years, and a BMI below 22 kg/m^2^ and aged >70 years [25]. Oncological information consisted of cancer type, size, local nodes, and metastasis; tumor-related information was converted to cancer stages 1–4 based on guidelines, to enable comparison between the different cancer types [26,27,28,29]. Any scans with streak artifacts at L3 related to high-density objects were excluded (e.g., osteosynthesis material or neurostimulators), due to their lack of clinical relevance in muscle segmentation. Manual segmentations for the included CT slices were performed by an experienced anatomist at the central level of L3 using MIM software (Version 7.0.1). All CT slices were processed according to the nnU-Net pipeline [22].

### 2.3. Inference Processing

The segmentations based on the external validation scans provided by the trained nnU-Net model were post processed in two steps. First, because L3 segmentation only includes trunk muscles, a DBSCAN was applied to exclude segmented arm muscles for individuals who positioned their arms alongside their body [30], a representative visualization is provided in Supplementary Figure S1. The DBSCAN parameters were set with an epsilon of 10 and a minimum sample size of five, meaning that a cluster was defined as a group of at least five pixels, with a maximum distance of 10 pixels in between. The centrally located cluster identified by DBSCAN was selected, thus excluding arm muscles.

Secondly, segmentation detail varied significantly between SAROS and the reference standard datasets, illustrated in Figure 1. Most differences in segmentation accuracy are attributed to the inclusion of adipose tissue and fascia, which have lower Hounsfield Unit (HU) values. To refine model segmentation accuracy, pixels below a specified HU threshold were excluded to remove adipose tissue and fascia. The optimal threshold between -60 HU and 0 HU was experimentally determined based on the highest overall median DSC derived from comparing the reference standard with the segmentation across all segmentations.

### 2.4. Analysis

Muscle surface areas from both the reference standard and model inference were summarized using the median and interquartile range (IQR). To evaluate agreement between the two, a Bland–Altman plot was employed, applying the conventional ±1.96 standard deviations to determine the upper and lower limits of agreement.

Since direct surface area comparisons may overlook geometric discrepancies in segmentation, the DSC (Formula (1)), quantifying the relative overlap between the reference and model segmentations, was calculated using SciPy [31]. To enhance clinical interpretability, SSE (cm^2^) (Formula (2)), measuring the non-overlapping surface, was calculated between the model predictions and the reference standard [32,33]. For each slice, SSE was calculated by summing all false-positive and false-negative pixels in the model segmentation. This count was then multiplied by the pixel size in cm^2^ obtained from the DICOM header to determine the misclassified surface area.

(1) $$\text{DSC}=1-\frac{\text{False Positive Pixels}+\text{False Negative Pixels}}{2*\text{True Positive Pixels}+\text{False Positive Pixels}+\text{False Negative Pixels}}$$

(2) $$\text{SSE}=(\text{False Positive Pixels}+\text{False Negative Pixels})*\text{Pixels Size}\;({\mathrm{cm}}^{2})$$

To assess any trends in factors influencing model performance, a visual inspection was performed on segmentations below the 25th DSC percentile. Due to their differences in size and differentiability, the visual inspection focused on six muscle groups: psoas major, quadratus lumborum, erector spinae, abdominal wall, rectus abdominis, and diaphragm, all highlighted in Supplementary Figure S2 [18]. To evaluate recurring segmentation errors, each error was classified as either involving non-muscle anatomy or having ill-defined boundaries. The prevalence of these two error types between underweight and non-underweight participants was calculated using the Fisher exact test.

Spearman’s rank correlation was used to examine a linear relationship between model performance metrics and both age and BMI. Correlation strength was interpreted as follows: coefficients < 0.40 were considered weak, 0.40–0.69 moderate, and ≥0.70 strong. The Mann–Whitney U test was employed to test for differences in performance for categorical variables such as gender, use of IV contrast, classified as underweight or not, and arm positioning (up vs. down). The model performance of the segmentation was analyzed across the different cancer types and tumor grades using the Kruskal–Wallis test, supplemented with Mann–Whitney U testing to assess differences between individual cancer types. The threshold for significance was set to *p* < 0.05 for all tests.

## 3. Results

**Figure 2:**
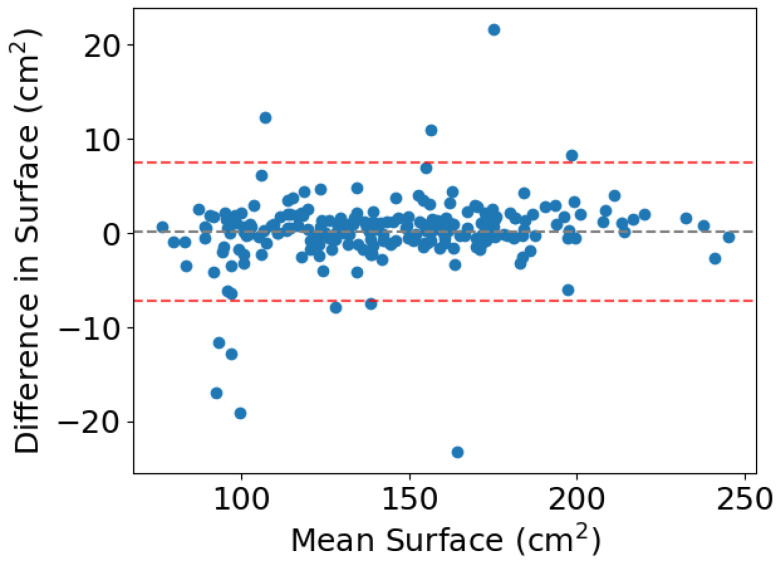
Bland–Altman plot illustrating the agreement between model-predicted and reference muscle surface areas. Mean difference: 0.119 cm^2^; limits of agreement: +7.488 cm^2^ and −7.250 cm^2^.

Automated segmentation was performed at L3 on all 232 scans from the external validation dataset. The resulting segmentations were compared to the reference standard by DSC and deviation in muscle surface. Experiments determining the optimal threshold between −60 HU and 0 HU indicated the highest median DSC at a level of −29 HU. Intermediate median DSC and HU thresholds are visualized in Supplementary Figure S3. Applying this threshold, the segmentations provided by the AI model trained using nnU-Net and the SAROS dataset resulted in a median muscle surface (IQR) of 140.181 cm^2^ (114.141–168.342 cm^2^), compared to 140.209 cm^2^ (116.315–169.265 cm^2^) for the reference segmentations. The mean difference in surface between the reference standard and model inference is 0.119 cm^2^ (Figure 2). In total, four and seven datapoints fall outside the Blant–Altman upper and lower limits of agreement of 7.488 cm^2^ and −7.250 cm^2^, respectively.

**Figure 3:**
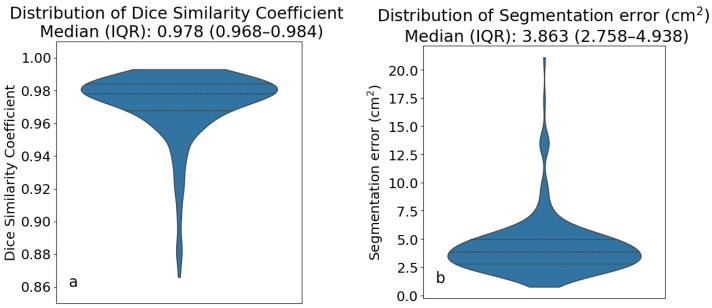
Violin plots showing the distribution of Dice Similarity Coefficient (**a**) and SSE (**b**) between model and reference segmentations. Horizontal dashed lines represent the median and quartiles.

Further geometric analysis shows a median (IQR) DSC of 0.978 (0.968–0.984) (Figure 3a), and SSE between the reference standard and model muscle segmentations with a median (IQR) of 3.863 cm^2^ (2.758–4.938 cm^2^) (Figure 3b).

### 3.1. Visual Inspection of Segmentation Errors

**Figure 4:**
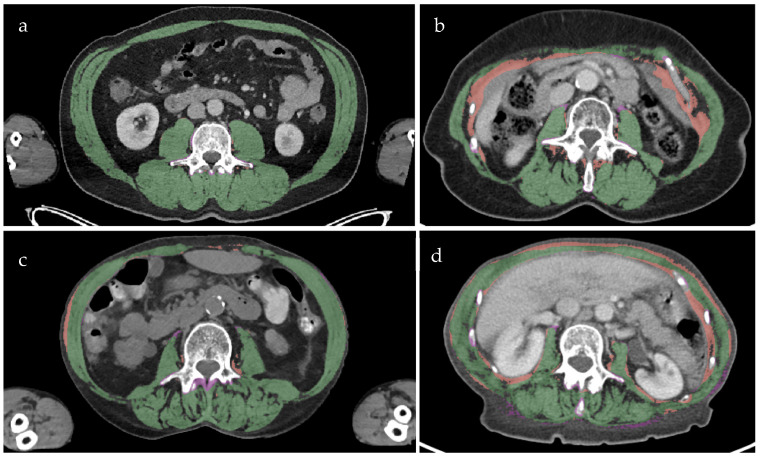
Model segmentation compared to the reference standard for non-underweight subjects with a DSC of 0.993 (**a**) and 0.900 (**b**), underweight subjects with a DSC of 0.973 (**c**) and 0.880 (**d**). All images are representative of segmentation performance regarding DSC for underweight and non-underweight subjects. Overlapping or true positive segmentation is green. False-positive segmentations (red), and false-negative segmentation (purple) highlight model errors.

Qualitative assessment of the lowest quartile DSC cases (*n* = 58, of which 17 were underweight subjects) indicated that segmentation errors were predominantly false positives (Figure 4). The abdominal wall muscles and rectus abdominis were associated with ±70% and ±30% of segmentation errors, respectively, followed by the psoas major in ±10%. The quadratus and lumborum muscles were involved in <5% of segmentation errors, and <7% of cases included the diaphragm. Ill-defined muscle boundaries were associated with 39 cases of segmentation errors, the inclusion of non-muscle anatomy with 21 cases, and four cases reported both segmentation error classes. Segmentation errors across underweight and non-underweight subjects were not significantly related to the inclusion of non-muscle anatomy (*p* = 1.000) or ill-defined muscle boundaries (*p* = 0.341).

### 3.2. Subject Characteristic Analysis

Statistical analysis regarding subject characteristics was conducted on 189 subjects, as 43 subjects from the complete cohort were excluded due to missing data. Population characteristics are shown in Table S1; missing values per variable are detailed in Table S2. The mean (SD) age of the subjects included for further analysis was 65 (±11) years old, 73% of subjects were male, and the remaining 27% were female. The mean (SD) BMI was 26 kg/m^2^ (±5 kg/m^2^), and 24 subjects were classified as underweight. The median (IQR) muscle surface of the underweight subjects was 104.081 cm^2^ (93.973–122.787 cm^2^), compared to 147.934 cm^2^ (124.950–172.424 cm^2^) for those not classified as underweight. Reported cancer types were melanoma (*n* = 69), lung (*n* = 56), esophageal (*n* = 41), and head and neck cancer (HNC) (*n* = 23). The distribution of underweight subjects across cancer types was unequal: melanoma (*n* = 2; 2%), lung (*n* = 7; 13%), esophageal (*n* = 7; 16%), and HNC (8; 27%).

**Figure 5:**
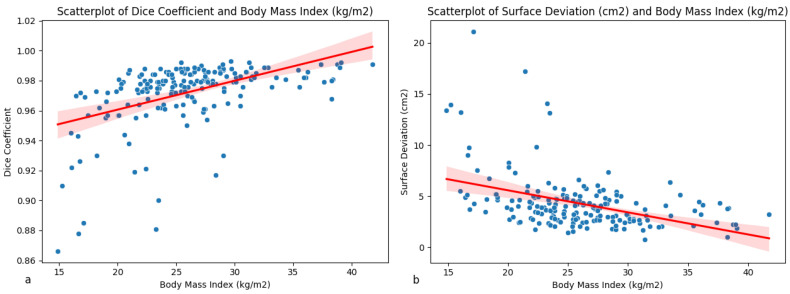
Scatterplots indicating the linear correlation between BMI and DSC (**a**) and BMI and SSE (**b**). Red indicates linear regression, with shading indicating 95% confidence interval.

Both DSC and SSE show a significant moderate correlation with BMI based on Spearman correlation coefficients of 0.517 (*p* < 0.05) and 0.406 (*p* < 0.05), respectively (Table 1). Scatterplots indicate the largest deviations at lower BMI values (Figure 5a,b).

Underweight subjects showed significant differences in DSC compared to non-underweight subjects, based on the Mann–Whitney U test (*p* < 0.001). Median (IQR) DSC are 0.979 (0.973–0.985) and 0.955 (0.932–0.969) for underweight and non-underweight subjects. The median SSE between underweight and non-underweight subjects showed a significant difference (*p* < 0.001) with 10.034 cm^2^ (8.419–14.694 cm^2^) and 6.094 cm^2^ (4.387–8.211 cm^2^), respectively.

A significant difference between male and female subjects was reported for DSC (*p* = 0.036), while no significant difference was found for SSE. The median (IQR) DSC for male and female subjects are 0.976 (0.959–0.981) and 0.979 (0.972–0.986), respectively.

Finally, a small but significant difference in DSC between cancer types was observed with the largest median difference in DSC of 0.006 between melanoma and HNC (*p*-value = 0.037). Pairwise comparison between cancer types resulted in *p*-values < 0.05 for individuals with melanoma compared to both individuals with HNC and with lung cancer (Table S3). No significant differences were identified between cancer types based on SSE (*p* = 0.488).

## 4. Discussion

This study aimed to provide an external validation of an AI model trained with nnU-Net on the SAROS dataset using CT images acquired during cancer diagnostics. The final model provides valid segmentation of muscles at the L3 level from CT images, achieving a mean surface difference of 0.119 cm^2^ compared to the reference standard. Bland–Altman analysis emphasized this finding with relatively narrow limits of agreement (7.488 cm^2^ and −7.250 cm^2^). The high median DSC of 0.978 also indicates minimal geometric discrepancies between model and reference segmentations for the whole population and subgroups alike. To our knowledge, the promising generalizability of openly available training pipelines and datasets for muscle segmentation through external validation with subgroup analysis has not been highlighted before.

The DSC obtained in our study exceeds the DSC reported in most prior muscle segmentation studies. Individual studies by Kreher et al. and Islam et al. reported DSC values < 0.95 [15,18,20]. Systematic reviews by Bedrikovetski et al. and Mai et al. corroborated these findings, yielding pooled DSCs of 0.941 and 0.942, respectively [16,17]. Higher segmentation performance is reported by Dietz et al., and Dabiri et al. report a DSC of 0.99, both using the same commercial platform. External validation, specifically focusing on subgroups, is limited for these studies.

Subgroup analyses tested variations in model performance based on DSC and SSE across subpopulations. Factors such as age, cancer grade, arm positioning, the use of intravenous contrast, and CCI did not impact segmentation performance defined by DSC and SSE. Sex showed a significant difference in DSC for males and females. Since the DSC depends heavily on the number of true-positive pixels, which correspond to the segmented muscle surface, it is likely impacted by the difference between sexes. Minimal clinical relevance of this finding is highlighted by the median DSC difference between male and female subjects of 0.003. An assumption that is further substantiated by a lack of significant difference in SSE representing a non-overlapping surface. The clinical impact of cancer types on DSC performance, though significant in two cases, is minimal. No significant difference in segmentation performance is reported for SSE. It is likely that performance differences between cancer types are caused by BMI differences amongst those cancer types.

BMI has a moderately significant correlation with both DSC and SSE. Furthermore, the model performed significantly worse for underweight individuals compared to non-underweight individuals. This finding is substantiated by the scatterplots indicating higher deviations for lower BMI values.

Segmentation errors are largely related to the inclusion of non-muscle anatomy and ill-defined muscle boundaries. However, no significant difference between underweight and non-underweight subjects is reported. Nonetheless, underweight subjects are more prone to overestimation of muscle surface, which may result in vulnerable individuals being incorrectly assessed as having adequate muscle surface. Potentially delaying necessary interventions such as (p-)rehabilitation [3,34]. Although the overall performance of the trained model is encouraging, we urge researchers to consider subgroup analysis and consider factors related to muscle quality, such as malnutrition, to assess model performance on vulnerable subjects. The described misclassification issues also underscore the limitations of rigid threshold-based classification systems. A more nuanced approach is essential, incorporating clinical context and including changes in muscle mass and subject history. Rigorous evaluation is essential to ensure safe and equitable integration of AI models into clinical workflows.

Our observations show that over two-thirds of segmentation errors occur in the abdominal wall muscles, while nearly one-third are found in the rectus abdominis. Inconsistent contrast and limited differentiability from surrounding tissue and muscles provide challenges for these muscle groups as described in the literature [18,20]. Although these muscles represent only a small portion of the total musculature at the L3 vertebral level, commonly seen as the reference standard for body composition analysis, their segmentation accuracy remains critical. How these observations translate to other vertebral levels or muscle volume remains unclear due to the L3 focus of this study. The choice to focus on L3 was based on conventional approaches; hence, volumetric assessment falls outside the scope of this study.

A key strength of this study lies in adopting HU thresholding as an accessible processing step to optimize segmentation accuracy. The public dataset used contained segmentations less detailed than the reference standard, often including adipose tissue within muscle regions. While such broader segmentations may align with certain clinical protocols, they compromise precision in muscle quantification. By applying an internally derived HU threshold to exclude adipose tissue, this study improved the specificity of muscle segmentation and brought model performance closer to the reference standard. This highlights the importance of segmentation detail, as it directly influences the validity of downstream analyses. Researchers and clinicians should therefore carefully evaluate segmentation granularity when adopting AI models for muscle assessment.

The adopted approach to HU thresholding could introduce an overfit, and optimization for individual subject characteristics is an option. However, Zoabi et al. reported only a weak significant correlation between BMI and HU, while no correlation was reported for age, weight, or height [35]. Hence, it is unlikely that patient characteristics will have a clinically relevant impact on the HU threshold, an assumption which is substantiated by the minimal, if not non-significant, findings between subgroups. In contrast, scan parameters will likely have a notable impact on the intended HU threshold. As reported by van der Werf et al., variations in tube voltage and the use of IV contrast will impact tissue HU [36]. Further in-depth assessment falls outside the scope of this study; however, we suggest a follow-up study to test these hypotheses.

From a clinical perspective, arm muscles are usually omitted from segmentation. However, depending on the imaging protocol and the patient’s condition, the arms may be placed alongside the torso. This is associated with streak artifacts, but our evaluation showed that these artifacts did not significantly impact model performance. This lack of significant difference suggests some robustness to variations in image quality related to arm positioning. However, subsequent studies should systematically evaluate the impact of image quality to ensure model generalizability across the diverse imaging conditions encountered in clinical practice.

Pathological and socio-demographic factors influence muscle composition [6]; therefore, two key limitations of the study population should be noted. All participants had a history of cancer, so the findings may not fully apply to non-cancer individuals. Nevertheless, the absence of significant differences across cancer types and BMI groups suggests a minimal clinical impact of these factors. Validation amongst other pathological backgrounds is needed to confirm this.

In addition, the study focused solely on individuals of Western European heritage due to limited ethnic diversity within the available patient pool. We intentionally prioritized a well-defined cohort and transparency over presenting an insufficiently diverse sample. Broader external validation is essential for assessing potential challenges when applying AI models beyond Western Europe [37]. While such analyses fall outside the scope of this work, we underline that our model is openly accessible and we welcome collaborations centered on diverse validation.

## 5. Conclusions

Muscle segmentation using nnU-Net, SAROS and HU-thresholding results in high external validity on an independent dataset. The use of publicly available data and AI tools presents a promising, cost-effective approach for researchers seeking to enhance segmentation speed and quality. However, the reduced model performance in underweight individuals highlights a key limitation. Further research is needed to assess this vulnerable group and assess generalizability to subjects of non-Western European descent.

## Figures and Tables

**Table 1:** Results from statistical analysis between model segmentations and reference standard based on performance metrics, DSC, and surface deviation. Significant differences between both segmentations are highlighted with ‘*’.

	Test ^1^	Dice Similarity Coefficient		Segmentation Surface Error	
		Correlation or Lowest DSC	*p*-Value	Correlation or Largest SSE	*p*-Value
Age (years)	Sp	corr. = −0.008	0.912	corr. = −0.123	0.092
BMI (kg/m^2^)	Sp	corr. = 0.517	<0.001 *	corr. = 0.406	<0.001 *
GLIM underweight (y/n) ^2^	MW	0.955	<0.001 *	5.195 cm^2^	<0.001 *
Sex (M/F)	MW	0.978	0.036 *	3.917 cm^2^	0.498
Cancer grade (1–4)	KW	0.977	0.125	4.191 cm^2^	0.136
Arm pos. (up vs. down) ^3^	MW	0.978	0.180	3.929 cm^2^	0.353
Use of IV (y/n)	MW	0.978	0.165	3.921 cm^2^	0.210
CCI (1–4)	KW	0.965	0.777	5.183 cm^2^	0.567
Cancer types	KW	0.974	0.037 *	5.902 cm^2^	0.488

^1^, Sp, Spearman. MW, Mann–Whitney U. KW, Kruskal–Wallis. ^2^, Classified as underweight based on GLIM-classification. ^3^, Arm positioning up, or along the head, and down, alongside the body.

## Data Availability

The SAROS dataset used in this study is publicly available at https://www.cancerimagingarchive.net/analysis-result/saros/ (accessed on 7 January 2025), while the nnU-net pipeline is available through https://github.com/MIC-DKFZ/nnUNet (accessed on 7 January 2025). Due to restrictions, the data used for external validation are not publicly available. However, the Python (version 3.10.4) code employed in this study does not introduce novel methods and can be provided upon reasonable request to the corresponding author.

## Supplementary Materials

The following supporting information can be downloaded at: https://www.mdpi.com/article/10.3390/jimaging12030135/s1. Figure S1: DBSCAN example; Figure S2: Visualization of the five included muscle groups; Figure S3: HU-thresholding visualization; Table S1: Subpopulation demographics; Table S2: Number of missing values per variable; Table S3: *p*-Values resulting of Mann–Whitney U analyses.

## Abbreviations

The following abbreviations are used in this manuscript:

AI	Artificial Intelligence
BMI	Body Mass Index
CT	Computed Tomography
DBSCAN	Density-Based Clustering Algorithm
DSC	Dice Similarity Coefficient
HU	Hounsfield Unit
IV contrast	Intravenous Contrast
L3	Lumbar Vertebra 3
nnU-Net	Open-Source AI Workflow
PET-CT	Positron Emission Tomography—Computed Tomography
SAROS	Sparsely Annotated Region and Organ Segmentation, a publicly available dataset
