# Supplementary material for: External Validation of an Open-Source Model for Automated Muscle Segmentation in CT Imaging of Cancer Patients
Source: J Imaging. 2026 Mar 18;12(3):135. doi: 10.3390/jimaging12030135 (PMC13028208; doi:10.3390/jimaging12030135)
Supplement: Supplementary file 1 [file jimaging-12-00135-s001.zip › Figures.pdf]

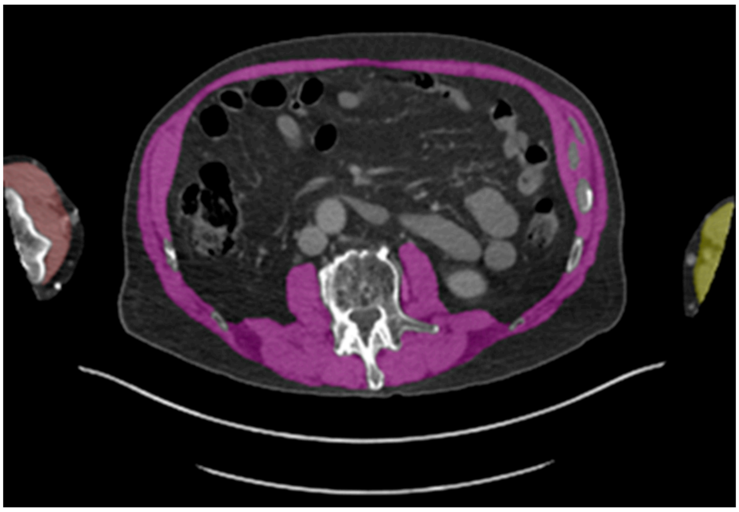

Figure S1: Example showing the separation of the arm muscles (red and yellow) from trunk muscles (purple) using DBSCAN.

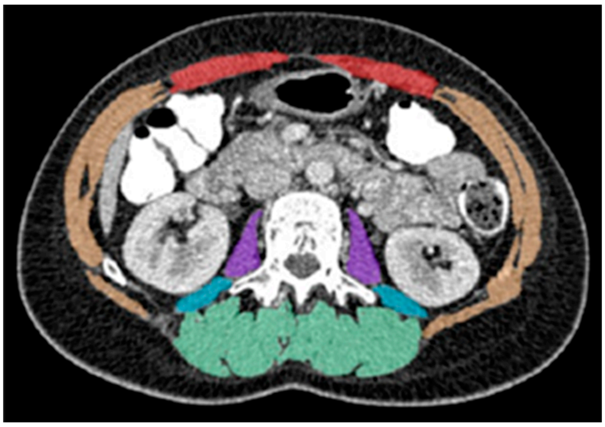

Figure S2: The five muscle groups used for visual inspection due to their difference in size and differentiability; Psoas major (purple), quadratus lumborum (blue), erector spinae (green), abdominal wall muscles (orange), rectus abdominis (red).

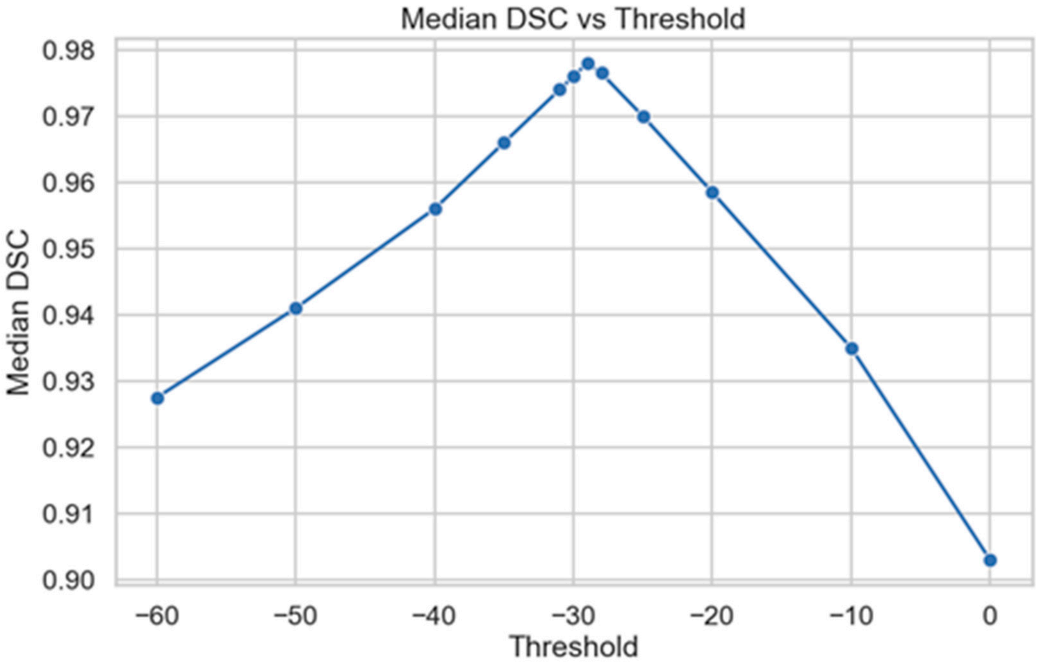


Figure S3: Median DSC for per HU-threshold, a threshold of -29 resulted in the highest median DSC and was used in post-processing of the segmentations provided by nnU-Net model.
