# Supplementary material for: External Validation of an Open-Source Model for Automated Muscle Segmentation in CT Imaging of Cancer Patients
Source: J Imaging. 2026 Mar 18;12(3):135. doi: 10.3390/jimaging12030135 (PMC13028208; doi:10.3390/jimaging12030135)
Supplement: Supplementary file 1 [file jimaging-12-00135-s001.zip › Table_S1.pdf]

Table S1: Characteristics of the patient subpopulation, including all variables used to assess the impact of patient attributes on model performance.

		Mean (SD) or n
Total included		189
Age (year)		65.249 (10.761)
Sex (m/f)		138/51
Arm position (arms up vs. arms down)		100/89
BMI (kg/m^2)		25.863 (5.243)
Use of IV (y/n)		158/31
CCI (1-4)		4.016 (2.295)
Underweight (n)		24
Cancer type	Melanoma	69
	Lung	56
	Oesophageal	41
	Head and neck	23
Cancer stage	1	23
	2	41
	3	69
	4	56
