# Supplementary material for: External Validation of an Open-Source Model for Automated Muscle Segmentation in CT Imaging of Cancer Patients
Source: J Imaging. 2026 Mar 18;12(3):135. doi: 10.3390/jimaging12030135 (PMC13028208; doi:10.3390/jimaging12030135)
Supplement: Supplementary file 1 [file jimaging-12-00135-s001.zip › Table_S2.pdf]

Table S2: Number of missing values per variable leading to the exclusion subjects in the final analysis.

Variable	Number of missing values
Age	5
Sex	5
BMI	8
Type of cancer	3
CCI score	19
Total number of subjects excluded, corrected for multiple missing values.	43
