# Supplementary material for: External Validation of an Open-Source Model for Automated Muscle Segmentation in CT Imaging of Cancer Patients
Source: J Imaging. 2026 Mar 18;12(3):135. doi: 10.3390/jimaging12030135 (PMC13028208; doi:10.3390/jimaging12030135)
Supplement: Supplementary file 1 [file Table_S3.pdf › Table_S3.pdf]

Table S3: Median (IRQ) DSC per cancer type and p-Values resulting of Mann-Whitney U analyses to determine significant differences in DSC between cancer types. The absolute differences between median DSC are presented in brackets behind the p-value, significant differences are highlighted using '*'.

	HNC (n=30)	Melanoma (n=96)	Lung (n=54)	Esophageal (n=45)
Median (IQR) DSC	0.974 (0.958-0.983)	0.980 (0.973-0.986)	0.978 (0.966-0.985)	0.976 (0.964-0.980)
p-value (Δ median DSC) Mann-Whitney U analyses cancer types				
HNC		0.046* (0.006)	0.681 (0.002)	0.372 (0.004)
Melanoma			<0.001* (0.02)	0.192 (0.004)
Lung				0.344 (0.002)
